# The Observer

**Published:** 1896-03

**Authors:** 


THE OBSERVER. A monthly journal of Natural Science. E. F. Bigelow, Publisher, Portland, Conn. Subscription price, $1.00 per year; 10 cents a single copy.

The March number of this elegant periodical is now before us. It is new to our book table, and so we have been giving it some extra attention.

It deals with all manner of scientific studies, but especially that of the microscope. The March number contains the following articles among others from which a very good idea may be obtained of its general character:

The Chimneys of Burrowing Crayfish, by R. W. Shufeldt, M. D., illustrated. Spring Buds, a suggestive study for the use of Teachers, by Miss E. E.

Carlisle. The Smallest Known Insect, by E. F. Bigelow, illustrated. Popular Astronomy, by Mary Proc'or. How I Became an Astronomer, by Mary Proctor. Mira, the Wonderful, by Garrett P. Serviss. The Canals of Mars, from Percival Lowell's articles. The Humpbacked Elves, by Elizabeth G. Britton.

These articles serve to show that the journal has a wide range of scientific thought, and is useful not only to physicians who have but scant leisure for outside reading, but also for the children and other members of their families.

